# Two predictive precision medicine tools for hepatocellular carcinoma

**DOI:** 10.1186/s12935-019-1002-z

**Published:** 2019-11-14

**Authors:** Zhiqiao Zhang, Jing Li, Tingshan He, Yanling Ouyang, Yiyan Huang, Qingbo Liu, Peng Wang, Jianqiang Ding

**Affiliations:** 1 Department of Infectious Diseases, Shunde Hospital, Southern Medical University, Shunde, Guangdong China; 2 Department of Hepatobiliary Surgery, Shunde Hospital, Southern Medical University, Shunde, Guangdong China

**Keywords:** mRNA, Hepatocellular carcinoma, Overall survival, Prognosis, Nomogram

## Abstract

**Background:**

Hepatocellular carcinoma (HCC) is a serious threat to public health due to its poor prognosis. The current study aimed to develop and validate a prognostic nomogram to predict the overall survival of HCC patients.

**Methods:**

The model cohort consisted of 24,991 mRNA expression data points from 348 HCC patients. The least absolute shrinkage and selection operator method (LASSO) Cox regression model was used to evaluate the prognostic mRNA biomarkers for the overall survival of HCC patients.

**Results:**

Using multivariate Cox proportional regression analyses, a prognostic nomogram (named Eight-mRNA prognostic nomogram) was constructed based on the expression data of N4BP3, -ADRA2B, E2F8, MAPT, PZP, HOXD9, COL15A1, and -NDST3. The C-index of the Eight-mRNA prognostic nomogram was 0.765 (95% CI 0.724–0.806) for the overall survival in the model cohort. The Harrell’s concordance-index of the Eight-mRNA prognostic nomogram was 0.715 (95% CI 0.658–0.772) in the validation cohort. The survival curves demonstrated that the HCC patients in the high risk group had a significantly poorer overall survival than the patients in the low risk group.

**Conclusion:**

In the current study, we have developed two convenient and efficient predictive precision medicine tools for hepatocellular carcinoma. These two predictive precision medicine tools are helpful for predicting the individual mortality risk probability and improving the personalized comprehensive treatments for HCC patients. The Smart Cancer Predictive System can be used by clicking the following URL: https://zhangzhiqiao2.shinyapps.io/Smart_cancer_predictive_system_HCC_2/. The Gene Survival Analysis Screen System is available at the following URL: https://zhangzhiqiao5.shinyapps.io/Gene_Survival_Analysis_A1001/.

## Introduction

Hepatocellular carcinoma (HCC) is the sixth most common type of cancer and the third leading cause of cancer-related death, resulting in 841,080 new cases and 781,631 deaths worldwide in 2018 [1]. The majority of HCC patients are diagnosed at an advanced stage because HCC is usually asymptomatic at an early stage. Surgical resection remains the main therapy for the majority of HCC patients. However, only 30–40% of HCC patients could be cured by surgical resection [2]. The intrahepatic recurrence rates in the remnant liver were 60–80% within 10 years after liver resection [3]. The 5-year and 10-year overall survival rates were 46.5% and 15.2%, respectively, for HCC patients who underwent surgical resection [4]. A systematic review of 4197 HCC patients demonstrated that the actual 10-year overall survival rate was only 7.2% after surgical resection [5]. Therefore, it is important to monitor HCC patients with high mortality risk and adopt effective strategies to improve the therapeutic efficacy.

With the substantial development of high-throughput sequencing technology, numerous genes have been reported to be related to the prognosis of HCC patients [6–9]. Li et al. developed a three-gene prognostic signature to predict the prognosis of HCC patients [10]. Zhai et al. constructed a mRNA classifier to predict the prognosis of HCC patients [11]. Zhen et al. established an eight-microRNA model to predict the overall survival of HCC patients [12]. However, these models were not user friendly, and the results were difficult for patients to understand without professional medical knowledge. In addition, these previous prognostic models could only provide the overall prediction of survival for a particular group but could not provide an individual risk prediction. More importantly, the Cox proportional hazards regression analysis is not suitable for high-dimensional microarray data due to the low ratio of sample size and variable number [13]. The least absolute shrinkage and selection operator method (LASSO) Cox regression method has been recommended for high dimensional microarray data [14].

The nomogram, which is derived from proportional hazard function, has been used as a straightforward predictive chart to predict the prognosis for various cancers [15, 16]. The nomogram is convenient for assessing the individual risk probability without a complex formula. The nomogram can provide straightforward individual risk assessment scores and the corresponding risk probability, which are easy to understand for patients without medical knowledge. Therefore, the prognostic nomogram is necessary for the prediction of individual risk probability in HCC patients. To the best of our knowledge, the present study is the first to construct a prognostic predictive nomogram for overall survival of HCC patients based on the mRNA sequencing data.

In the present study, we identified the prognostic biomarkers for overall survival using the least absolute shrinkage and selection operator method (LASSO) Cox regression model. Subsequently, we developed and validated a prognostic nomogram to predict the overall survival of HCC patients.

## Patients and methods

### Study protocol approval

The downloading, analyses and utilization of study datasets in the present study were performed according to the relevant data policies of The Cancer Genome Atlas (TCGA) database and Gene Expression Omnibus (GEO) database. Ethics approval and informed consent are not required for the present study according to the public database guideline.

### Gene information for model cohort

The gene expression data of hepatocellular carcinoma patients in the model cohort were downloaded from the TCGA data portal (https://tcga-data.nci.nih.gov). The model dataset contained 24,991 mRNA expression data points from 371 cancer samples and 50 adjacent normal tissues. The mRNA expression data were generated on the Illumina HiSeq 2000 RNA Sequencing platform. The original mRNA expression values were directly downloaded from the TCGA database.

### Survival analyses

For the survival analyses, the clinical survival information of 376 HCC patients in the model cohort were downloaded from the cBioPortal database (http://www.cbioportal.org/data_sets.jsp). Twenty-eight patients were excluded from the present study according to the following criteria: (1) patients with clinical survival data but without mRNA expression data were excluded (n = 8), and (2) patients with incomplete prognostic information or overall survival < 1 month were excluded to avoid the impact of unrelated causes of death (n = 20). The missing data in the study dataset were handled by multiple imputation techniques based on a random forest algorithm, if necessary. The medians of mRNA expression values were used as cut-off values to stratify the mRNA expression values into the high expression group (as value 1) and low expression group (as value 0). The overall survival was calculated as the time from the initial diagnosis to the time of death or the last follow-up. The maximum follow-up time was 120.7 months, and the minimum follow-up time was 0.3 month. Additional file 1: Figure S1 presents the flowchart of patient selection.

### Differential gene expression analyses

The differential gene expression analyses were performed using 371 hepatocellular carcinoma samples and 50 adjacent normal liver tissues. The original mRNA expression count values were normalized with the “edgeR” package using the Trimmed Mean of M (TMM) method [17]. The F-tests were used for the assessment of quasi-likelihood. The criteria for differential gene expression analyses were 0.5-fold change for downregulation and twofold change for upregulation. The threshold of statistical significance was set at *P *< 0.05.

### Information for validation cohort

The present study identified the GSE14520 dataset, which consists of 203 HCC patients, as the validation cohort for the prognostic nomogram (https://www.ncbi.nlm.nih.gov/geo/query/acc.cgi?acc=GSE14520). The mRNA expression values were generated on the Affymetrix HT Human Genome U133A Array platform.

### Heat map and volcano plot

The heat map and volcano plot were generated for 371 hepatocellular carcinoma samples and 50 adjacent normal liver tissues using the “edgeR” and “gplots” packages. The darkness of the colour on the heat map represented the mRNA expression level: the darker the colour was, the higher the level of mRNA expression was.

### The least absolute shrinkage and selection operator method Cox regression

The least absolute shrinkage and selection operator method (LASSO) Cox regression model is a suitable and valuable method for high dimensional microarray data [14]. LASSO Cox regression performs a sub-selection of variables by shrinkage of the respective regression coefficient by imposing the penalty proportional to their size. Through the sub-selection of variables, LASSO Cox regression ultimately provides a relatively small number of variables with a weight that is different than zero [18]. Therefore, LASSO Cox regression can be used for optimal selection of high dimensional microarray data [19].

### Variable selection and prognostic model construction

The LASSO Cox regression was performed to identify the most informative prognostic mRNA biomarkers for the overall survival. Based on the optimal lambda value (11.363), 26 potential mRNA biomarkers were identified as potential prognostic biomarkers for overall survival. The final prognostic model was constructed with a multivariate Cox regression model (backward stepwise) based on the selected prognostic mRNA biomarkers. The prognostic nomogram for individual prediction of overall survival was constructed based on the results of the multivariate Cox regression model.

### Predictive performance of nomogram

The Harrell’s concordance index (C-index) was used to assess the predictive performance and discriminative ability of the prognostic nomogram. The calibration plot of the prognostic nomogram was performed to verify the concordance between the predicted probability and the observed probability. The time-dependent receiver operating characteristic (ROC) curves were conducted to access the discriminative ability of the prognostic nomogram for 1-year, 2-year and 3-year overall survival.

### Statistical analysis

Normal distribution continuous data were expressed as the mean ± standard deviation. The non-normal distribution data were expressed as the median (first quartile, third quartile). Continuous data were compared by t-test or Mann–Whitney U test as appropriate. Categorical data were compared by Chi-squared test or Fisher’s exact test as appropriate. The LASSO Cox regression was used to identify the informative biomarkers for overall survival. Thereafter, these potential biomarkers were entered into the multivariate Cox regression model to construct a predictive nomogram for overall survival. Kaplan–Meier survival analyses were used to generate and compare the survival curves of different risk groups. The differences between the survival curves of different risk groups were compared by the log-rank test.

The mRNA expression original values were normalized with the “edgeR” package. The Cox regression analyses were carried out using the “survival” package. The least absolute shrinkage and selection operator method (LASSO) Cox regression model was performed using “glmnet” package. The nomogram and calibration plot were conducted with “rms” package. Time-dependent receiver operating characteristic (ROC) curve was performed using “pROC” package. The analyses were carried out using R software (version 3.4.1) and SPSS Statistics 19.0 (SPSS Inc., an IBM Company). A two-tailed *P* value < 0.05 was considered to be statistically significant.

## Results

### Study cohorts

There were 348 and 203 HCC patients in the model cohort and validation cohort, respectively. All patients included in the present study had a pathological diagnosis of HCC. Overall, 130 (37.4%) patients died during the follow-up period in the model cohort, whereas 81 (39.9%) patients died in the validation cohort. The demographics and clinical characteristics of HCC patients in the model cohort and validation cohort are summarized in Table 1.

**Table 1:** The demographics and clinical features of hepatocellular carcinoma patients in model cohort and validation cohort

	Model cohort	Validation cohort	*P* value
Patients (n)	348	203	
Death [n (%)]	130 (37.4)	81 (39.9)	0.553
Survival time (month)	20.5 (11.9, 37.7)	51.3 (16.2, 57.3)	< 0.001
Age (year)	59.5 ± 13.4	51.1 ± 10.6	< 0.001
Male [n (%)]	236 (67.8)	174 (85.7)	< 0.001
Grade1 [n (%)]	53 (15.2)	NA	
Grade2 [n (%)]	163 (46.8)	NA	
Grade3 [n (%)]	115 (33.0)	NA	
Grade4 [n (%)]	12 (3.4)	NA	
AJCC PT1 [n (%)]	171 (49.1)	NA	
AJCC PT2 [n (%)]	87 (25.0)	NA	
AJCC PT3 [n (%)]	74 (21.3)	NA	
AJCC PT4 [n (%)]	14 (4.0)	NA	
AJCC PN0 [n (%)]	244 (70.1)	NA	
AJCC PN1 [n (%)]	3 (0.9)	NA	
AJCC PN2 [n (%)]	100 (28.7)	NA	
AJCC PM0 [n (%)]	248 (71.3)	NA	
AJCC PM1 [n (%)]	100 (28.7)	NA	
AJCC stage1 [n (%)]	164 (47.1)	83 (40.9)	0.033
AJCC stage2 [n (%)]	79 (22.7)	71 (35.0)	
AJCC stage3 [n (%)]	80 (23.0)	48 (23.6)	
AJCC stage4 [n (%)]	4 (1.1)	0	
Child–pugh1 [n (%)]	210 (60.3)	NA	
Child pugh2 [n (%)]	20 (5.7)	NA	
Child pugh3 [n (%)]	1 (0.3)	NA	
Radiation treatment [n (%)]	4 (1.1)	NA	
Pharmaceutical [n (%)]	15 (4.3)	NA	
Ablation embolization [n (%)]	13 (3.7)	NA	
Family history [n (%)]	106 (30.5)	NA	

The survival time was expressed as median (first quantile, third quantile). Continuous variables were compared by t-test or Mann–Whitney U test as appropriate. Categorical variables were compared by Chi-squared test or Fisher’s exact test as appropriate

### Differential expression of mRNAs

The study dataset of the model cohort consisted of 371 cancer samples and 50 adjacent normal tissues and included a total of 24,991 mRNA expression data points. Using “edgeR” package, we identified 436 differentially expressed mRNAs whose *P*-value was less than 0.05. Additional file 2: Figure S2 and Additional file 3: Figure S3 show the heat map and volcano plot for the differentially expressed mRNAs, respectively.

### Variable selection and identification of prognostic mRNA biomarkers

**Fig. 1:**
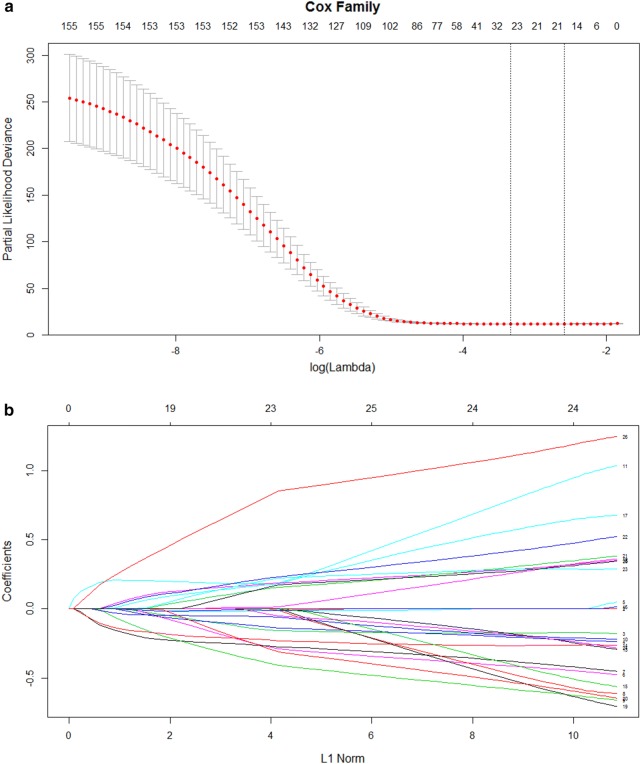
The selection of prognostic mRNAs determined by the least absolute shrinkage and selection operator Cox regression model. **a** Tuning parameter selection cross-validation error curve. The vertical lines were drawn according to the values of the minimum criteria and the 1-SE criteria. **b** The path of the coefficients of the 26 differentially expressed mRNAs included in the optimal model

The least absolute shrinkage and selection operator (LASSO) method was used to evaluate the most informative prognostic mRNA biomarkers according to their relative contribution to the prognostic model [20]. A cross-validated error plot of the LASSO Cox model is presented in Fig. 1a. According to the results of the LASSO Cox regression model with a lambda value of 11.363, 26 prognostic mRNAs with non-zero regression coefficients were finally chosen as the potential prognostic biomarkers for the overall survival of HCC patients (Fig. 1b).

### Construction of prognostic nomogram

**Fig. 2:**
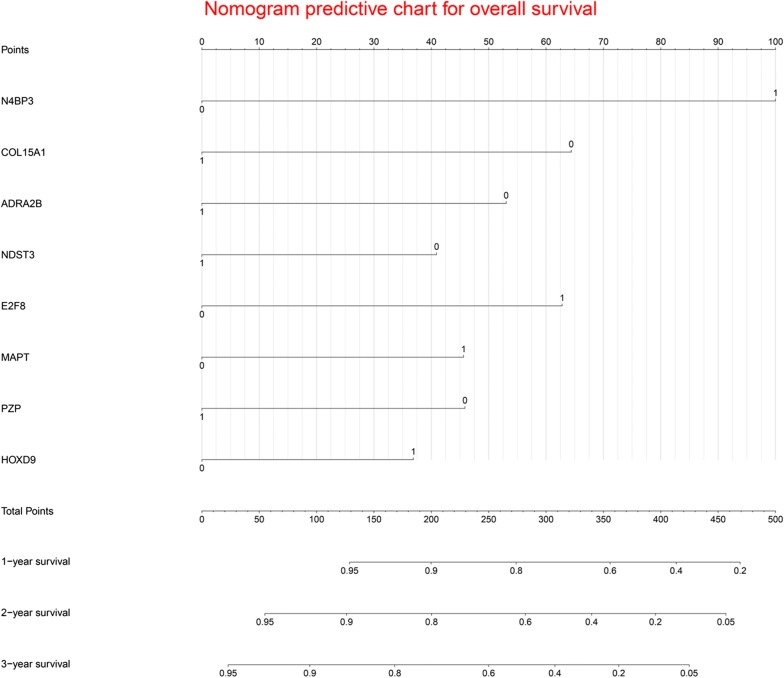
The nomogram to predict 1-year, 2-year and 3-year overall survival for hepatocellular carcinoma patients

Using multivariate Cox proportional regression (backward stepwise method), a prognostic nomogram (named Eight-mRNA prognostic nomogram) was constructed based on the potential prognostic predictors determined by the LASSO Cox regression model (Fig. 2). The coefficients derived from the Cox regression model are summarized in Table 2. The formula of the Eight-mRNA prognostic nomogram was as follows: Eight-mRNA prognostic nomogram score = (0.598525 * N4BP3) − (0.114211 * -ADRA2B) + (0.321434 * -E2F8) + (0.198411 * MAPT) − (0.216164 * PZP) + (0.171283 * HOXD9) − (0.005399 * -COL15A1) − (0.066424 * -NDST3). The mRNA expression values were translated into 0 for low expression and 1 for high expression, according to the median values of the mRNA expression values.

**Table 2:** The model information of prognostic mRNA biomarkers in univariate and multivariable Cox regression analyses

	Univariate analyses			Coefficient	Multivariate analyses		
	HR	95% CI	*P*-value		HR	95% CI	*P*-value
N4BP3 (high vs. low)	2.135	1.492–3.054	0.001	1.012	2.751	1.883–4.019	0.001
COL15A1 (high vs. low)	0. 38	0.378–0.765	0.001	− 0.652	0.521	0.358–0.760	0.001
ADRA2B (high vs. low)	0.471	0.330–0.671	0.001	− 0.537	0.585	0.399–0.856	0.006
NDST3 (high vs. low)	0.575	0.404–0.818	0.002	− 0.414	0.661	0.460–0.949	0.025
E2F8 (high vs. low)	2.033	1.425–2.898	0.001	0.635	1.888	1.295–2.752	0.001
MAPT (high vs. low)	1.875	1.315–2.672	0.001	0.462	1.587	1.101–2.288	0.013
PZP (high vs. low)	0.597	0.420–0.849	0.004	− 0.464	0.629	0.440–0.898	0.011
HOXD9 (high vs. low)	1.842	1.294–2.623	0.001	0.374	1.453	1.011–2.089	0.044

The median values of mRNA expression were used as cutoff values to stratify mRNA expression values into high expression group (as value 1) and low expression group (as value 0)

### Performance of the Eight-mRNA prognostic nomogram in the model cohort

**Fig. 3:**
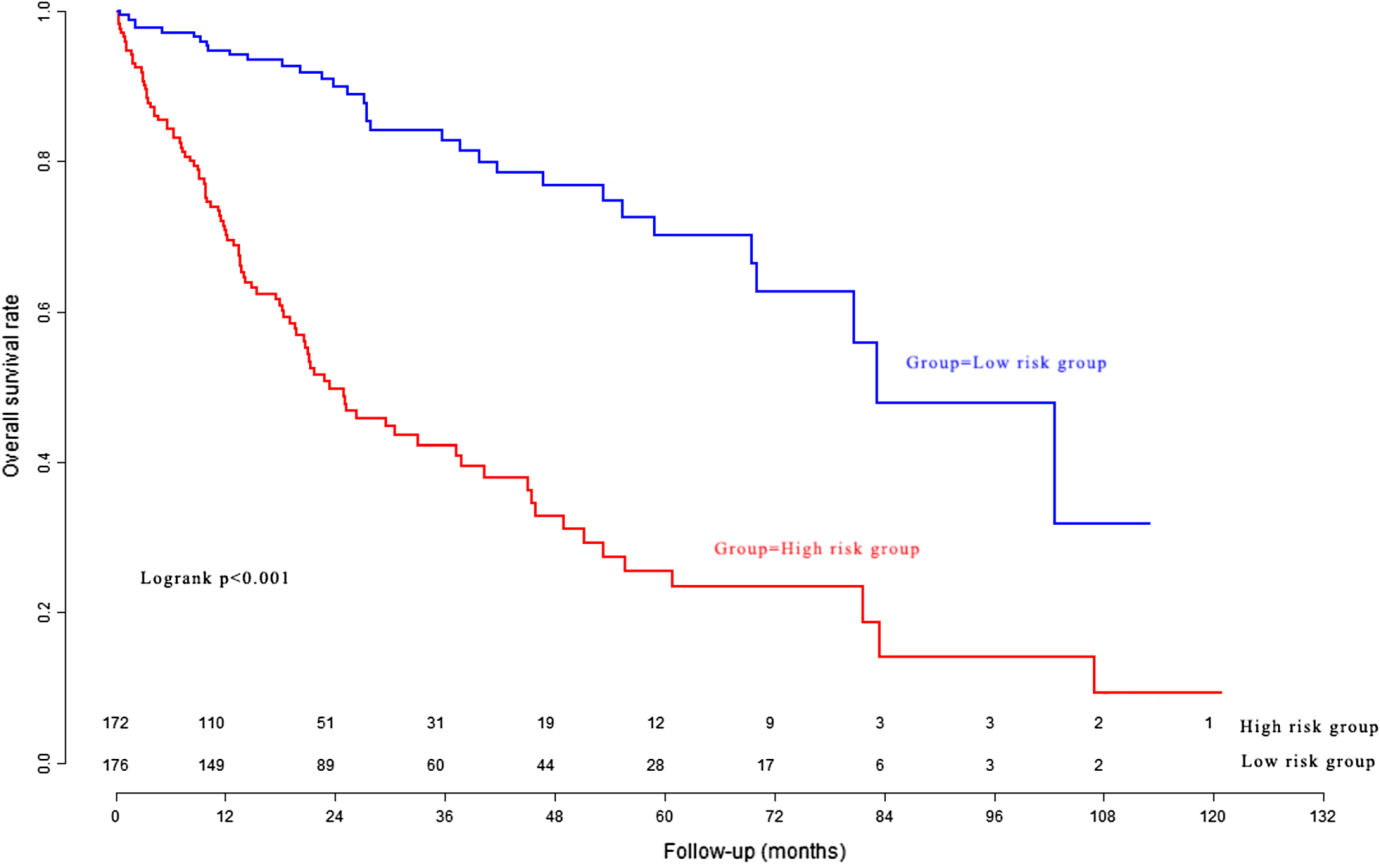
The overall survival curves of the high risk group and low risk group determined by nomogram in the model cohort

According to the Eight-mRNA prognostic nomogram scores, 348 HCC patients in the model cohort were divided into the high risk group and low risk group. The overall survival curves of the two groups are presented in Fig. 3. The patients in the high risk group had significantly poorer survival than the patients in the low risk group. In the model cohort, the Harrell’s concordance-index (C-index) was 0.765 (95% CI 0.724–0.806).

### Time-dependent receiver operating characteristic curves in the model cohort

**Fig. 4:**
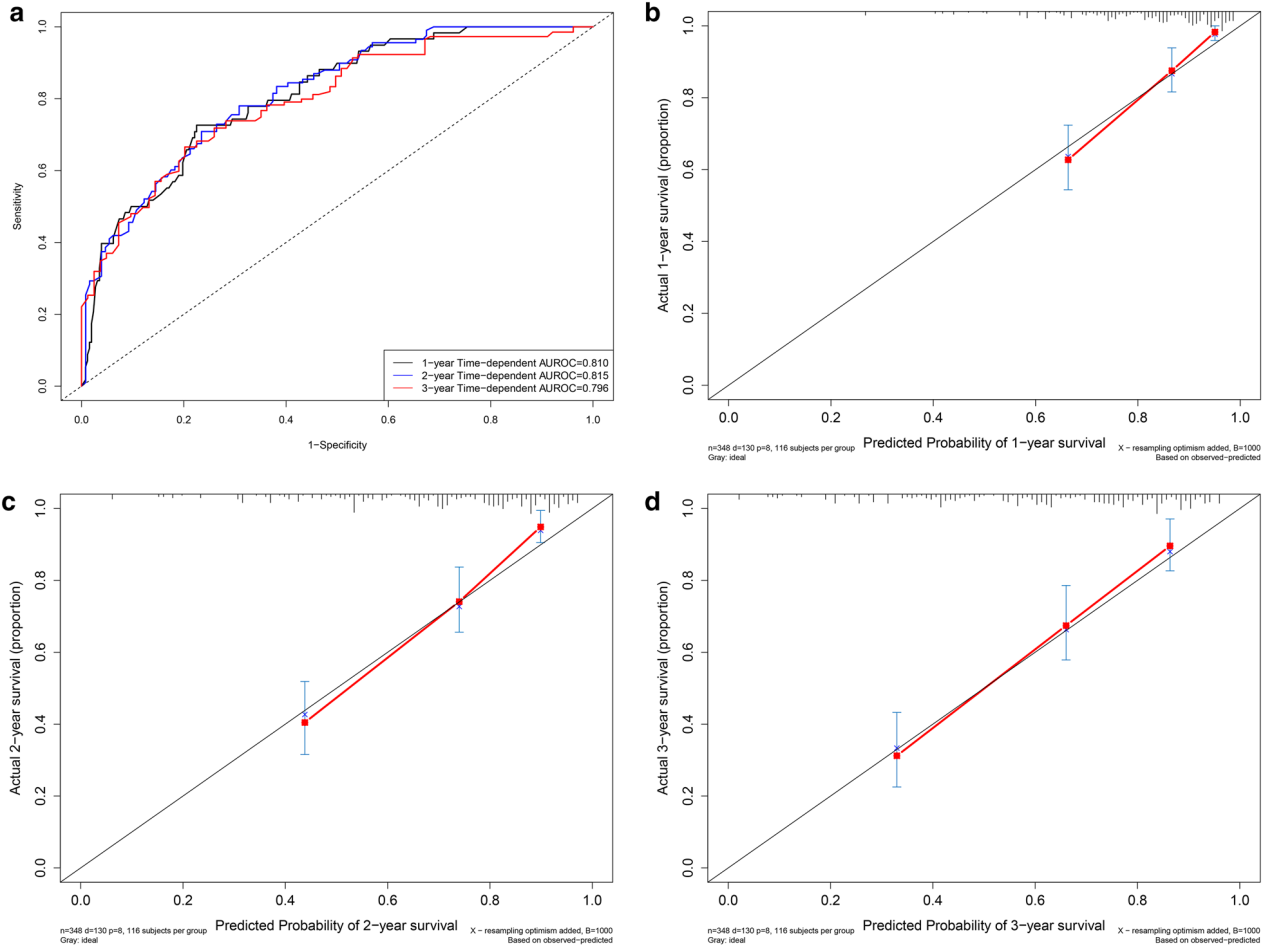
Performance of the nomogram in the model cohort. **a** Time-dependent receiver operating characteristic curves according to the 1-year, 2-year and 3-year overall survival status in the model cohort. **b** Calibration curves for 1-year overall survival. **c** Calibration curves for 2-year overall survival. **d** Calibration curves for 3-year overall survival

Time-dependent ROC curves were drawn according to the 1-year, 2-year and 3-year overall survival status in the model cohort (Fig. 4a). The C-indexes for 1-year, 2-year and 3-year overall survival were 0.810 (95% CI 0.769–0.851), 0.815 (95% CI 0.760–0.842) and 0.796 (95% CI 0.755–0.837), respectively.

### Calibration curves in the model cohort

The calibration curves for 1-year (Fig. 4b), 2-year (Fig. 4c) and 3-year (Fig. 4d) overall survival demonstrated that the actual survival probability was similar to the predicted survival probability.

### Clinical utility of the Eight-mRNA prognostic nomogram in the validation cohort

**Fig. 5:**
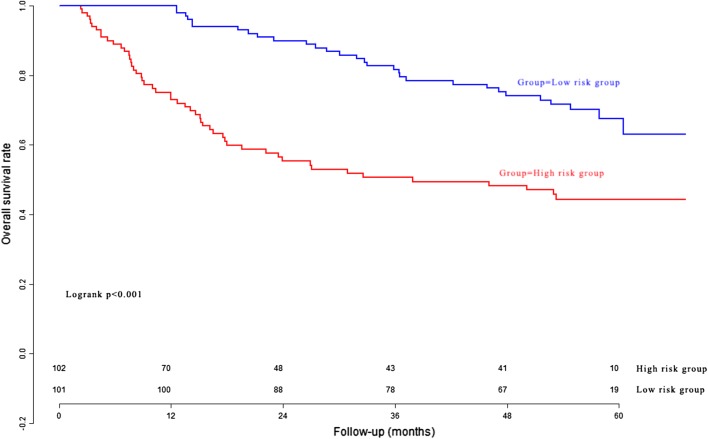
The overall survival curves of the high risk group and low risk group in the validation cohort

The Eight-mRNA prognostic nomogram scores in the validation cohort were generated according to the previous formula of the Eight-mRNA prognostic nomogram. The C-index of the Eight-mRNA prognostic nomogram was 0.715 (95% CI 0.658–0.772) for the validation cohort. The survival curves of different risk groups are presented in Fig. 5.

### Time-dependent ROC curves and calibration curves in the validation cohort

**Fig. 6:**
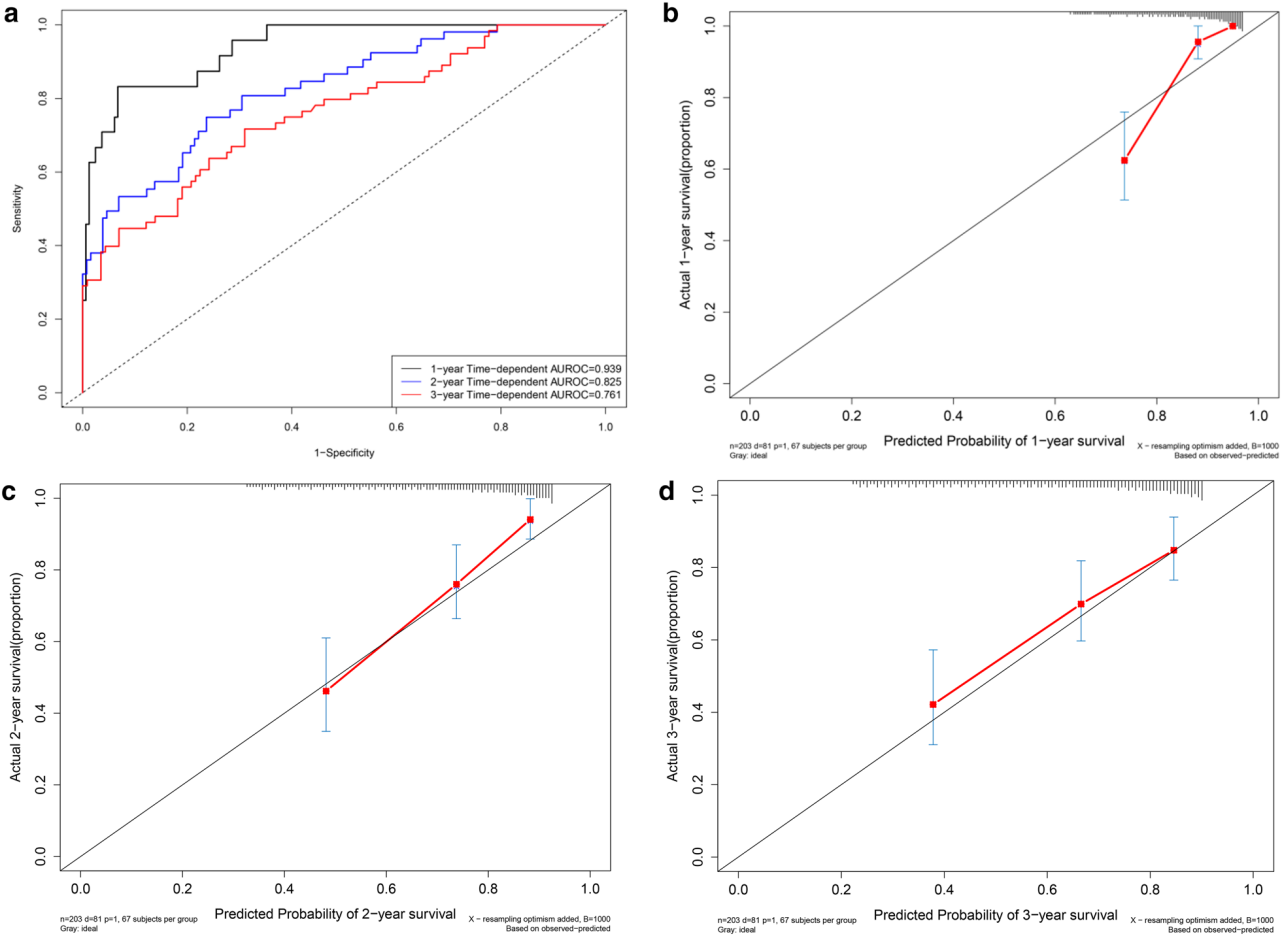
Performance of the nomogram in the validation cohort. Time-dependent receiver operating characteristic curves according to the 1-year, 2-year and 3-year overall survival status in the validation cohort (**a**). Calibration curves for 1-year overall survival (**b**). Calibration curves for 2-year overall survival (**c**). Calibration curves for 3-year overall survival (**d**)

In the validation cohort, the C-indexes for 1-year, 2-year and 3-year overall survival were 0.939 (95% CI 0.882–0.996), 0.825 (95% CI 0.768–0.882) and 0.761 (95% CI 0.704–0.818), respectively (Fig. 6a). The calibration curves for 1-year (Fig. 6b), 2-year (Fig. 6c) and 3-year (Fig. 6d) overall survival demonstrated that the actual survival probability was similar to the predicted survival probability.

### Survival curve analyses of prognostic mRNA biomarkers

**Fig. 7:**
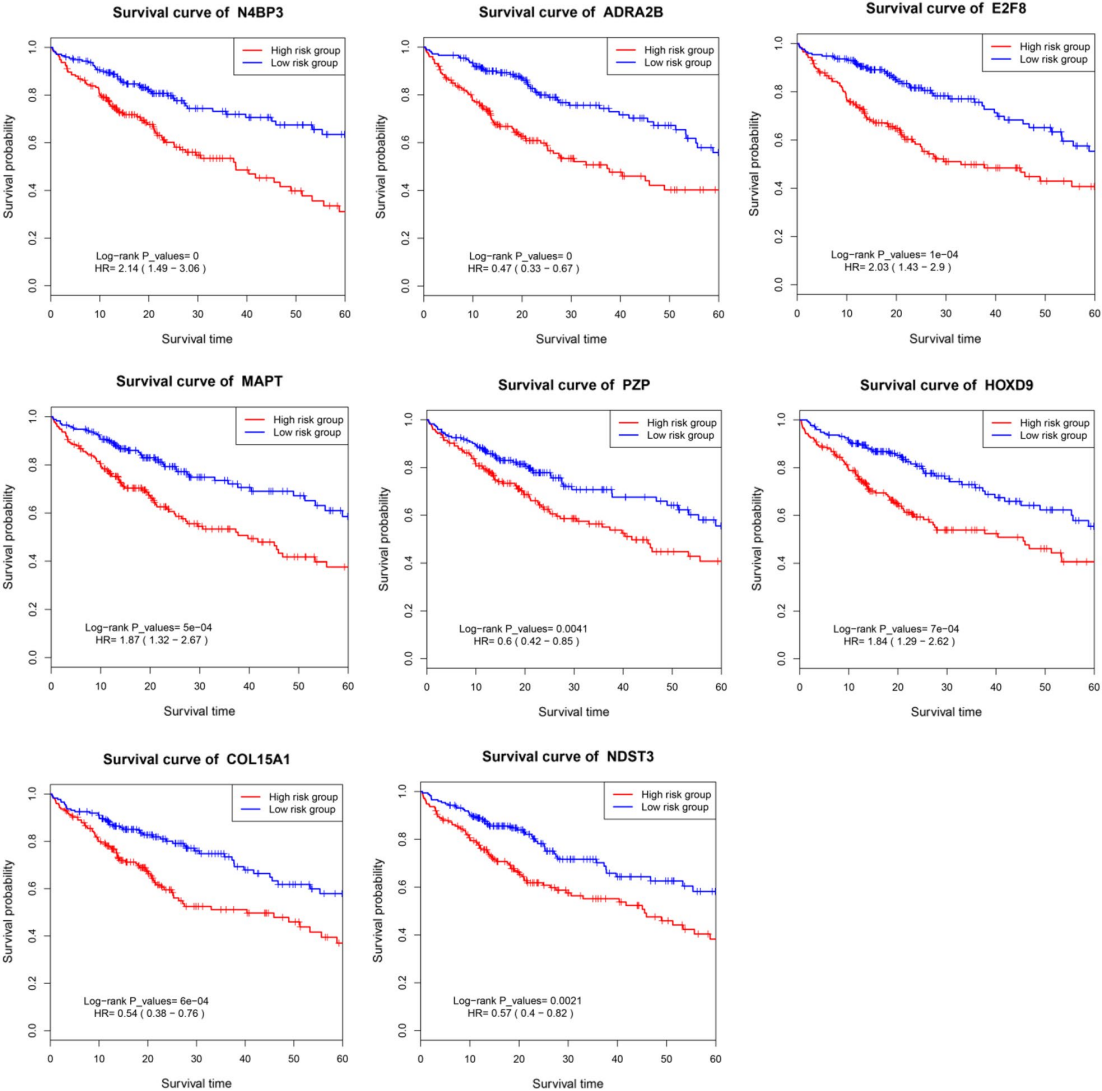
Survival curve analysis of mRNAs in the prognostic nomogram

The survival curve analyses of prognostic mRNAs in the Eight-mRNA prognostic nomogram are presented in Fig. 7. The overall survival rates were significantly different between the high risk group and low risk group for prognostic mRNA biomarkers in the Eight-mRNA prognostic nomogram (*P *< 0.001).

### Independence assessment of Eight-mRNA prognostic nomogram

As shown in Table 3, the Eight-mRNA prognostic nomogram and AJCC PM were the independent factors affecting the overall survival according to multivariate Cox regression analyses.

**Table 3:** Univariate and multivariable Cox regression analyses

	Univariate analyses			Coefficient	Multivariate analyses		
	HR	95% CI	*P*-value		HR	95% CI	*P*-value
Age (≥ 61 years vs. < 61 years)	1.347	0.950–1.910	0.094				
Gender (male vs. female)	0.817	0.573–1.164	0.264				
Grade (3–4 vs. 1–2)	1.118	0.780–1.603	0.545				
AJCC PT (3–4 vs. 1–2)	2.548	1.794–3.617	< 0.001				
AJCC PN (1–2 vs. 0)	1.516	1.052–2.185	0.026				
AJCC PM (1–2 vs. 0)	1.674	1.162–2.413	0.006	0.803	2.231	1.269–3.924	0.005
AJCC stage (3–4 vs. 1–2)	2.442	1.685–3.540	< 0.001				
Child pugh (2–3 vs. 1)	1.614	0.796–3.270	0.184				
Prognostic nomogram (high vs. low)	4.162	2.798–6.191	< 0.001	1.591	4.909	2.751–8.762	< 0.001

*AJCC* The American Joint Committee on Cancer, *HR* hazard ratio, *CI* confidence interval

### Subgroup analyses

**Fig. 8:**
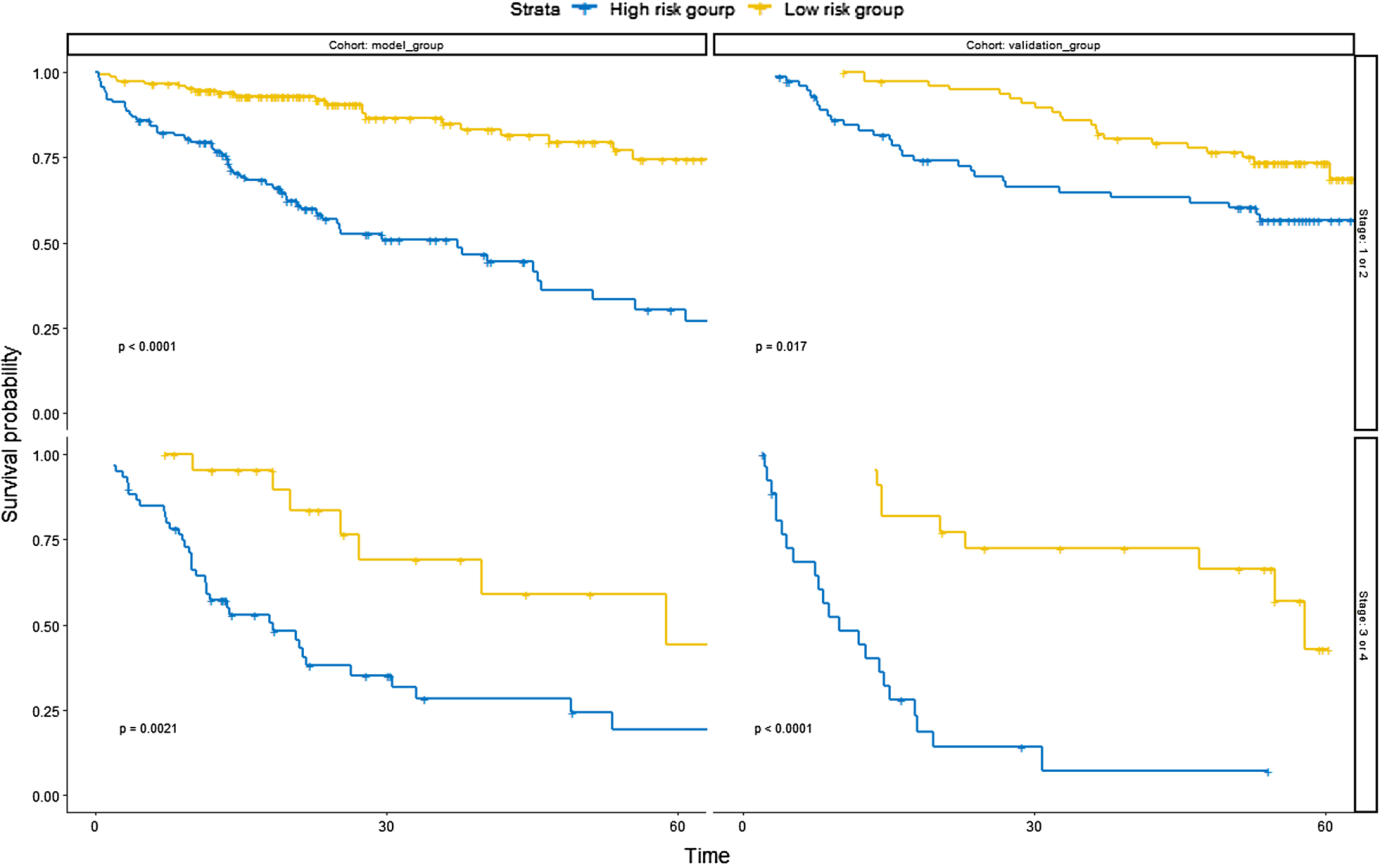
Survival curve analyses in different subgroups

Subgroup analyses (Fig. 8) indicated that the overall survival rates the in high risk group were significantly lower than those in the low risk group in the different cohorts and pathological stages.

### Gene expression using the immunohistochemical method

**Fig. 9:**
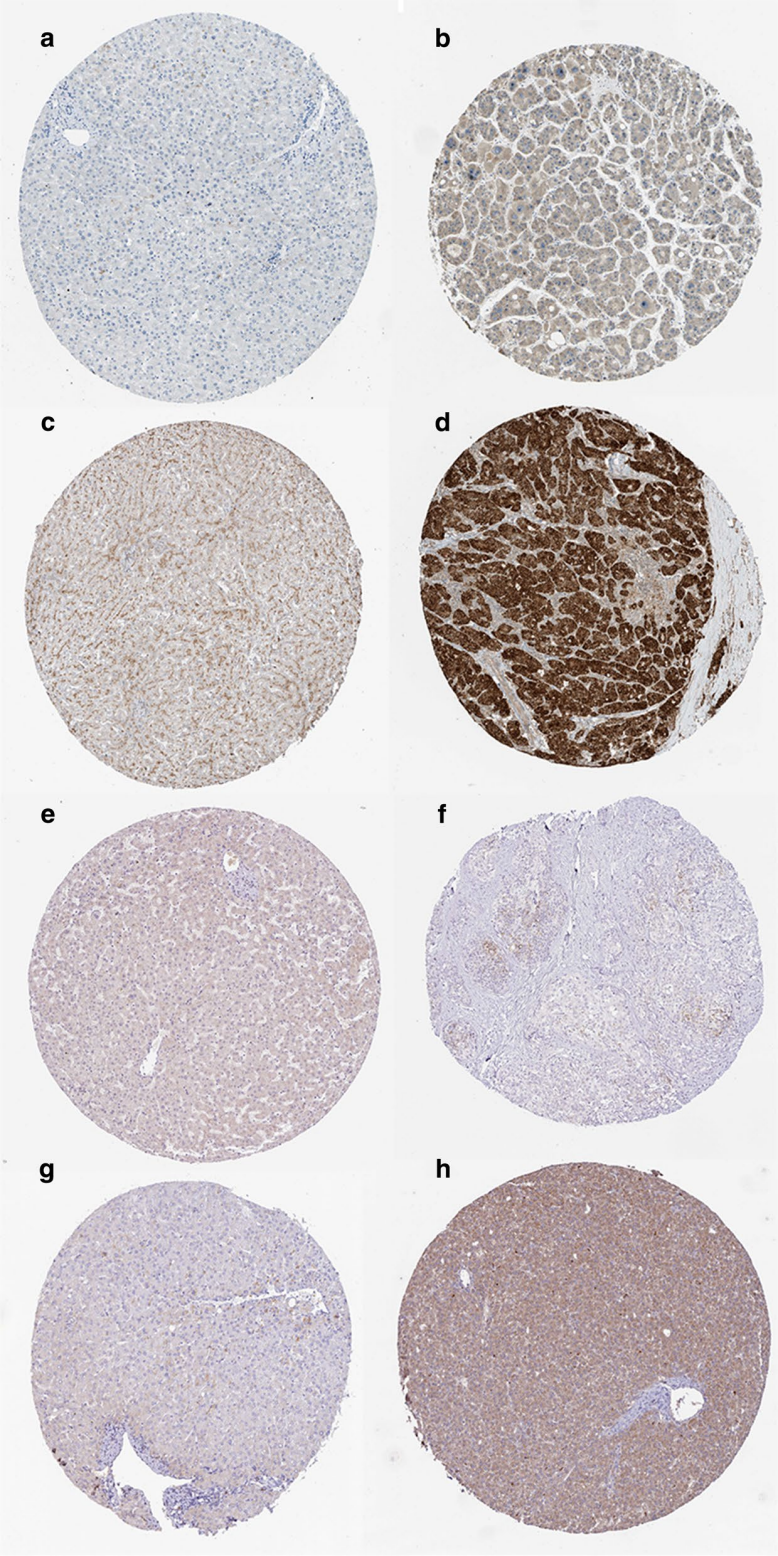
Gene expression in hepatocellular carcinoma samples and normal tissues by immunohistochemistry. **a** Negative expression of COL15A1. **b** Positive expression of COL15A1. **c** Negative expression of N4BP3. **d** Positive expression of N4BP3. **e** Negative expression of NDST3. **f** Positive expression of NDST3. **g** Negative expression of PZP. **h** Positive expression of PZP

The gene expression of eight prognostic mRNA biomarkers were assessed in the normal tissues and HCC specimens based on the Human Protein Atlas database (https://www.proteinatlas.org/). As shown in Fig. 9, the expression levels of COL15A1 (Fig. 9a for negative and Fig. 9b for positive), N4BP3 (Fig. 9c for negative and Fig. 9d for positive), NDST3 (Fig. 9e for negative and Fig. 9f for positive), and PZP (Fig. 9g for negative and Fig. 9h for positive) were significantly different between the normal tissues and HCC specimens.

### Correlation analysis between the prognostic genes and clinical parameters

**Fig. 10:**
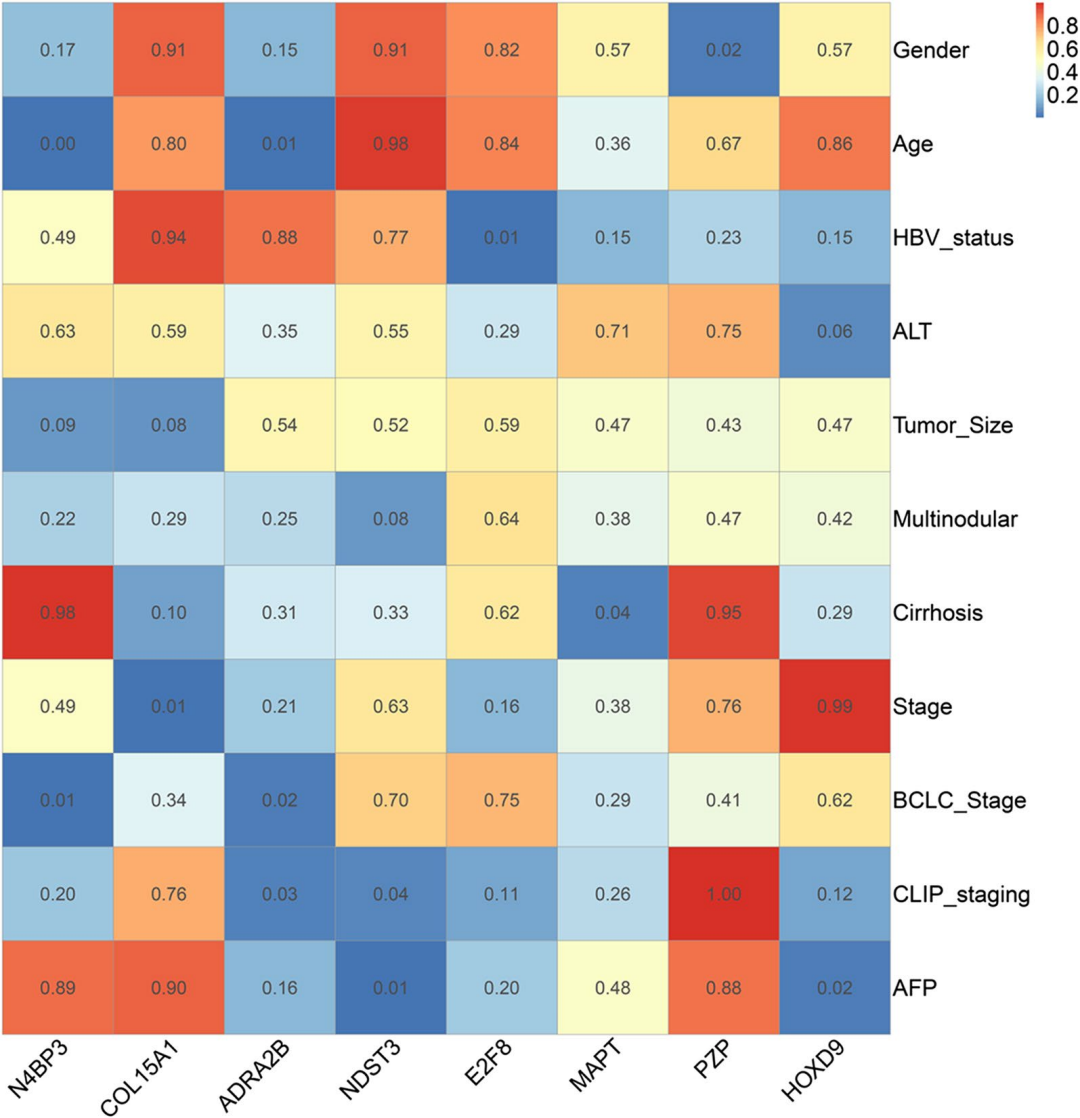
Correlation coefficient heatmap of mRNA biomarkers and clinical parameters

**Fig. 11:**
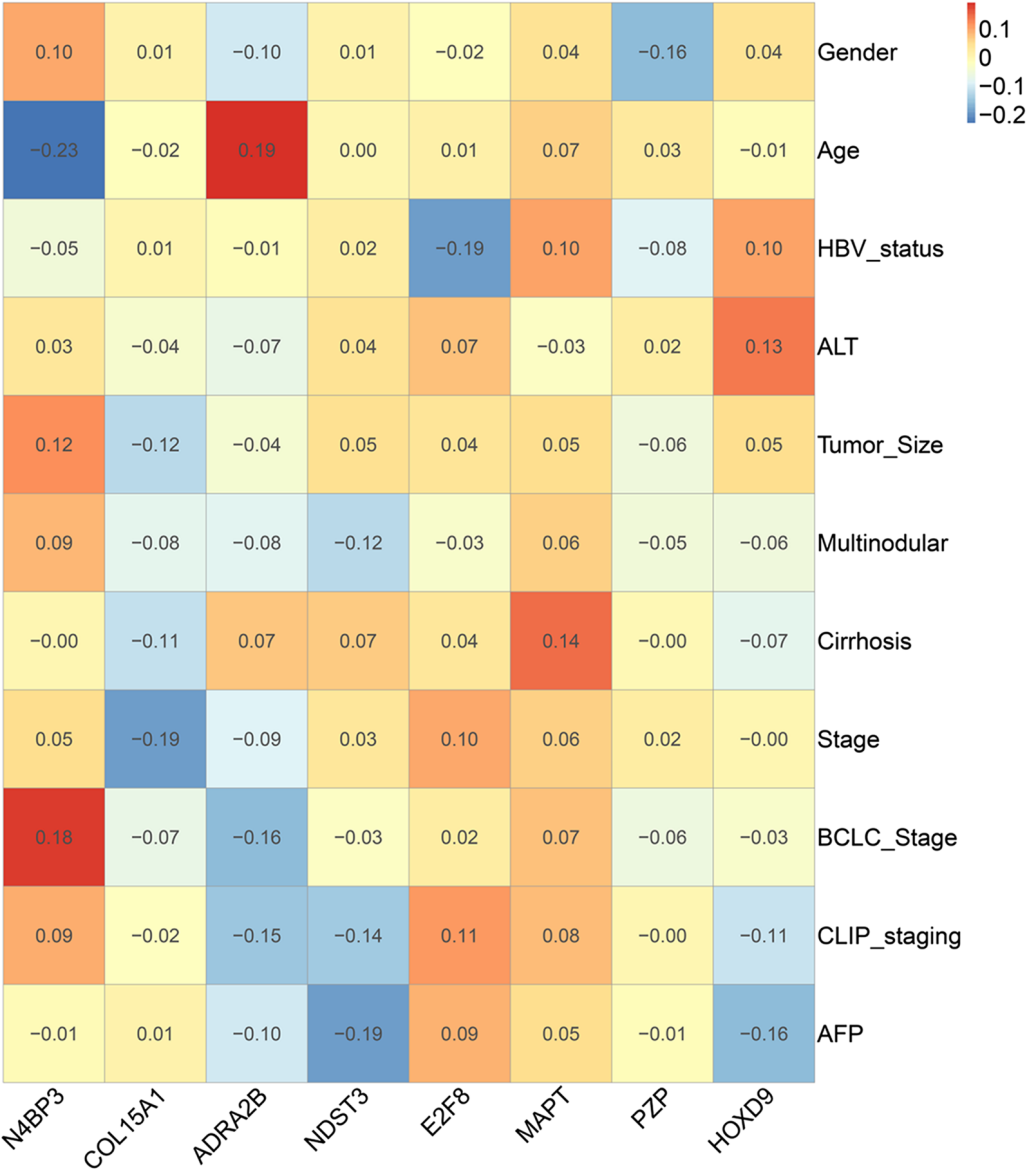
Correlation significance heatmap of mRNA biomarkers and clinical parameters

**Fig. 12:**
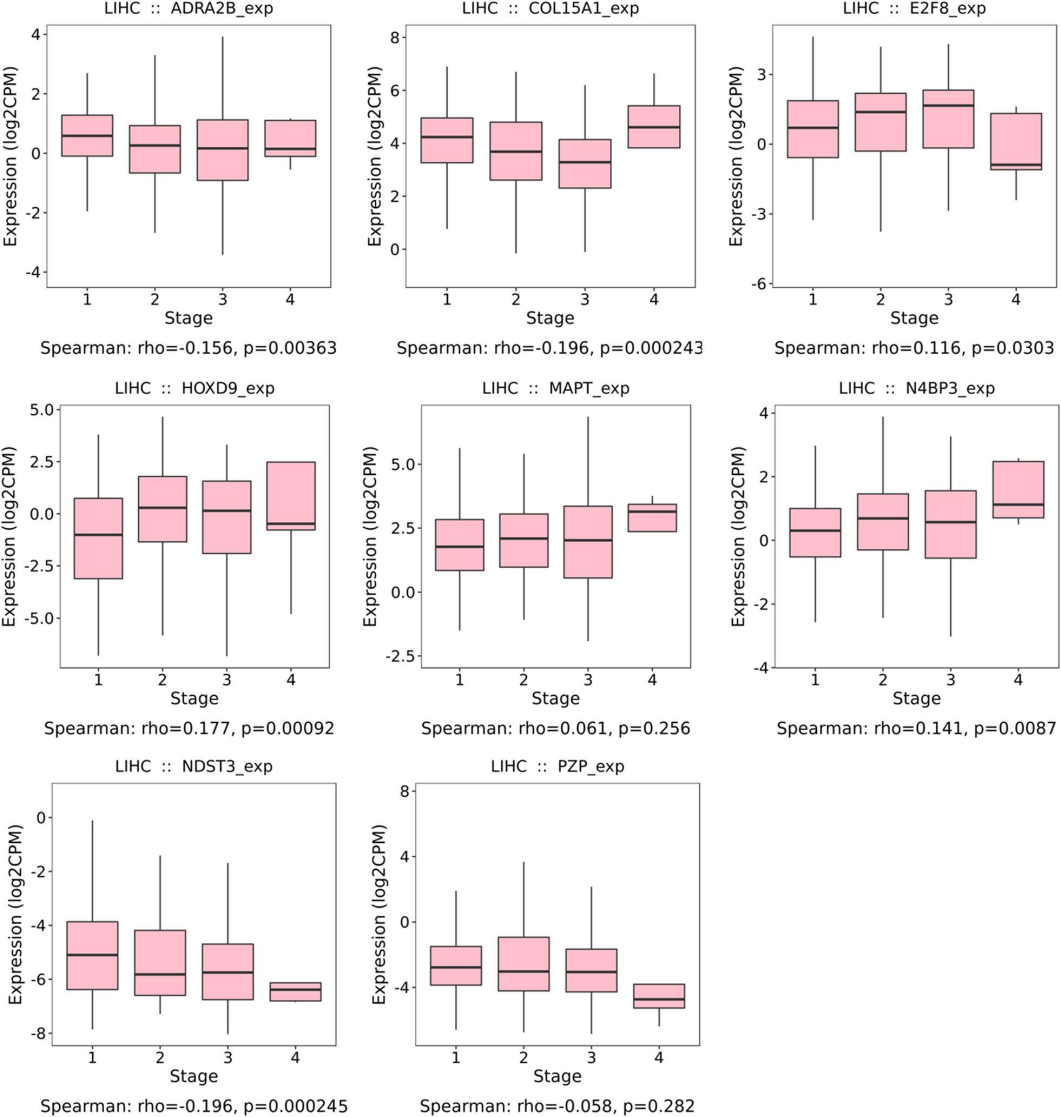
Expression levels of mRNA biomarkers in different pathological stages

To evaluate the correlation analysis between prognostic genes and clinical parameters, we constructed a correlation coefficient heatmap (Fig. 10) and a correlation significance heatmap (Fig. 11) for the mRNA biomarkers and clinical parameters. The distribution of the prognostic genes at the different pathological stages is presented in Fig. 12.

### Protein–protein interaction network

**Fig. 13:**
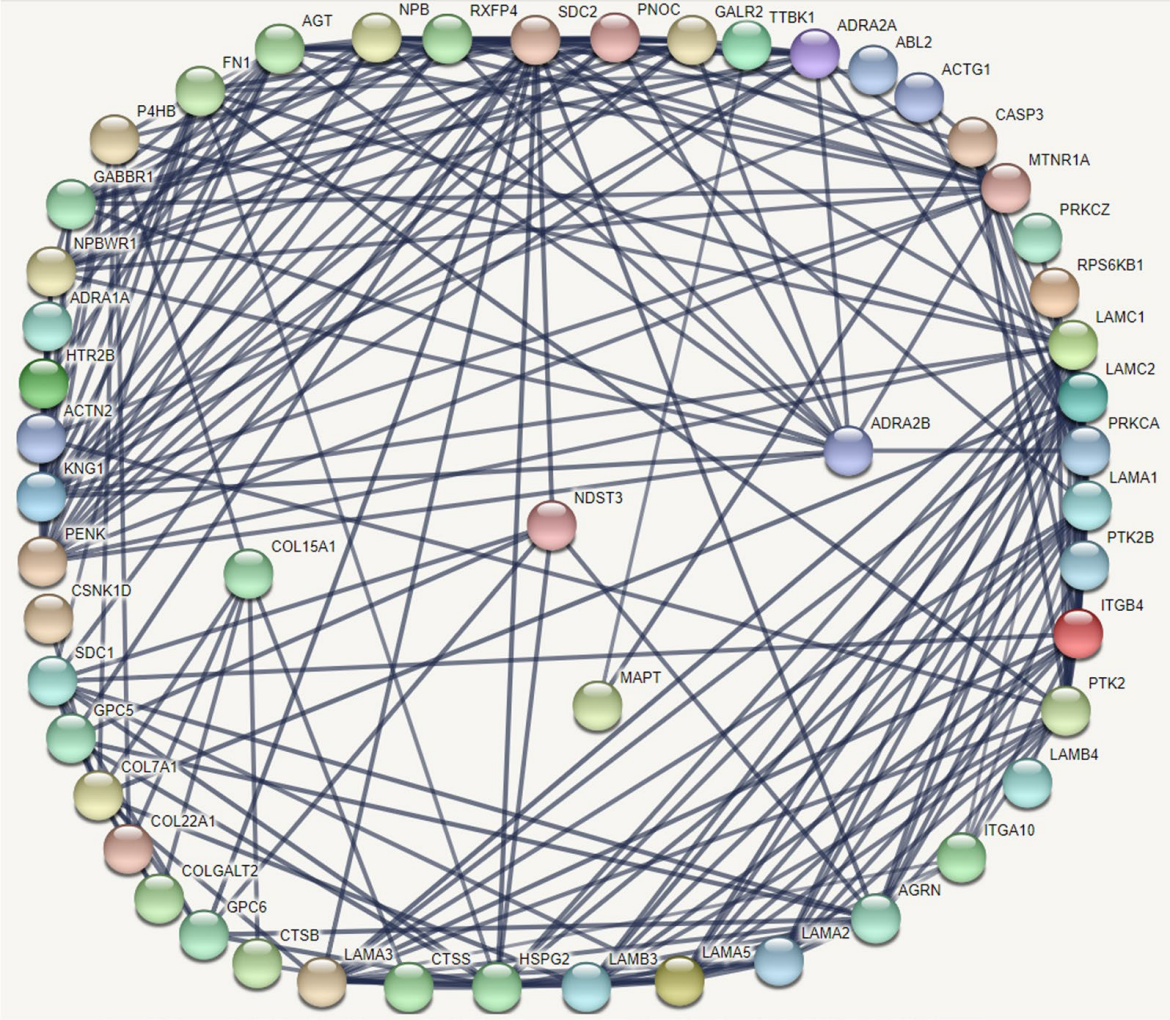
Protein–protein interaction network chart

To evaluate the potential association among the prognostic genes, we constructed the protein–protein interaction network (PPI) using the Search Tool for the Retrieval of Interacting Genes (STRING, https://string-db.org/) database (Fig. 13). The PPI network contained 51 nodes, including four prognostic genes and 47 most frequently altered neighbour genes.

### Cell line analysis

The cell line analysis was performed by RNA-seq to estimate the transcript abundance of each protein-coding gene according to the Human Protein Atlas database (https://www.proteinatlas.org/). The Cell Atlas provides RNA expression data derived from RNA sequencing of a large panel of cell lines and protein localization data derived from antibody-based profiling by immunofluorescence confocal microscopy, using a subset of cell lines selected based on RNA expression.

**Fig. 14:**
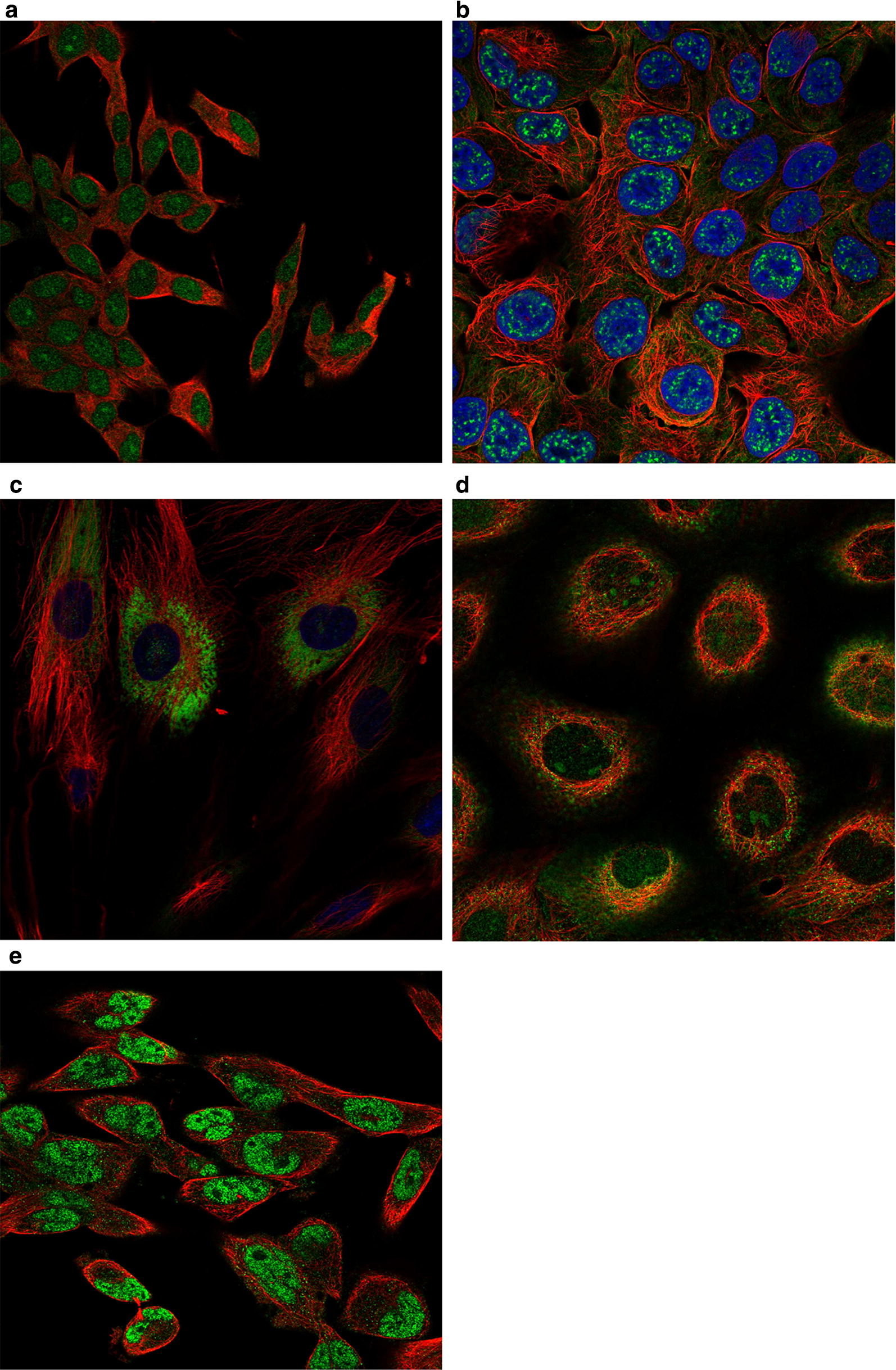
Cell line distribution chart: **a** HOXD9, **b** MAPT, **c** COL15A1, **d** E2F8, **e** N4BP3

As shown in Fig. 14, N4BP3 localized to the nucleoplasm and centrosome (Antibody: HPA030973; Cell line: RH-30; Cell line RNA Expression: 10.7.

Location: Nucleoplasm). E2F8 was detected in the nucleoli, nucleoplasm, and cytosol (Antibody: HPA064882; Cell line: A-431; Cell line RNA Expression: 14.2.

Location: Nucleoli and Cytosol). MAPT was detected in the plasma membrane and nuclear speckles (Antibody: HPA048895; Cell line: RT4; Cell line RNA Expression: 3.5; Location: Nuclear speckles and Plasma membrane).

HOXD9 was detected in the nucleoplasm and nucleoli (Antibody: HPA068683; Cell line: SH-SY5Y; Cell line RNA Expression: 16.4; Location: Nucleoplasm and Nucleoli). COL15A1 was detected in the endoplasmic reticulum (Antibody: HPA017913; Cell line: BJ; Cell line RNA Expression: 13.0; Location: Endoplasmic reticulum).

### Association between the prognostic mRNAs and hepatocellular carcinoma

We further explored the association between prognostic mRNA biomarkers and hepatocellular carcinoma using the Open Targets Platform database (https://www.targetvalidation.org/). The Open Targets Platform database integrated clinical evidence and provided overall scores for the association between the prognostic mRNAs and hepatocellular carcinoma. The overall association scores for hepatocellular carcinoma were 0.210 for HOXD9, 0.174 for NDST3, 0.111 for PZP, 0.106 for E2F8, 0.061 for ADRA2B, and 0.029 for COL15A1.

### Exploration of the survival curves in various subgroups

To further explore the survival curves of the previous prognostic genes in different sex and pathological stage subgroups, we developed a new online program named the Gene Survival Analysis Screen System. The Gene Survival Analysis Screen System is available at the following URL: https://zhangzhiqiao5.shinyapps.io/Gene_Survival_Analysis_A1001/.

## Discussion

Using LASSO Cox regression model, we constructed an Eight-mRNA prognostic nomogram to predict the overall survival of HCC patients. Our results demonstrated that the Eight-mRNA prognostic nomogram was helpful for estimating individual mortality risk and could identify HCC patients with high mortality risk. Time-dependent ROC curves and calibration curves demonstrated that the predictive performance of the Eight-mRNA prognostic nomogram was robust and reliable.

From the clinical practice perspective, the poor overall survival of HCC patients remains a serious challenge for public health management. The HCC patients in the high mortality risk group have a poor overall survival and should receive more active comprehensive treatments compared with the HCC patients in the low mortality risk group. Therefore, early identification and individual prediction are of importance for HCC patients with high mortality risk, and these patients should undergo timely appropriate comprehensive treatments. This Eight-mRNA prognostic nomogram is suitable to stratify the HCC patients according to mortality risk and, accordingly, to help the high risk patients receive timely treatments. To the best of our knowledge, this is the first prognostic nomogram to predict the overall survival of HCC patients based on their mRNA expression data.

The biological process of COL15A1 is mainly enriched in angiogenesis (GO:0001525), collagen catabolic process (GO:0030574), collagen metabolic process (GO:0032963), multicellular organism metabolic process (GO:0044236), and blood vessel morphogenesis (GO:0048514). The biological process of adrenoceptor alpha 2B (ADRA2B) is mainly enriched in activation of MAPK activity (GO:0000187), muscle system process (GO:0003012), circulatory system process (GO:0003013), vascular process in circulatory system (GO:0003018), muscle contraction (GO:0006936), and regulation of smooth muscle contraction (GO:0006940). N-deacetylase/N-sulfotransferase (heparan glucosaminyl) 3 (NDST3) is mainly enriched in aminoglycan metabolic process (GO:0006022), aminoglycan biosynthetic process (GO:0006023), glycosaminoglycan biosynthetic process (GO:0006024), proteoglycan metabolic process (GO:0006029), sulfur compound metabolic process (GO:0006790), and glycoprotein metabolic process (GO:0009100). NEDD4 binding protein 3 (N4BP3) plays a role in axon and dendrite arborization during cranial nerve development. The biological process of E2F transcription factor 8 (E2F8) is mainly enriched in cytokinesis (GO:0000910), angiogenesis (GO:0001525), in utero embryonic development (GO:0001701), liver development (GO:0001889), placenta development (GO:0001890), and embryonic placenta development (GO:0001892). The biological process of microtubule-associated protein tau (MAPT) is mainly enriched in microtubule cytoskeleton organization (GO:0000226), regulation of cell growth (GO:0001558), neuron migration (GO:0001764), autophagy (GO:0006914), microtubule-based movement (GO:0007018), and axonogenesis (GO:0007409). The biological process of pregnancy-zone protein (PZP) is mainly enriched in pregnant females (GO:0007565), negative regulation of peptidase activity (GO:0010466), negative regulation of endopeptidase activity (GO:0010951), multi-multicellular organism process (GO:0044706), negative regulation of proteolysis (GO:0045861), and negative regulation of hydrolase activity (GO:0051346). The biological process of homeobox D9 (HOXD9) is mainly enriched in skeletal system development (GO:0001501), regionalization (GO:0003002), single fertilization (GO:0007338), pattern specification process (GO:0007389), peripheral nervous system development (GO:0007422), and muscle organ development (GO:0007517).

Several mRNA biomarkers included in the Eight-mRNA prognostic nomogram have been reported as prognostic biomarkers for HCC patients in previous studies. Deng et al. reported that E2F8 contributed to the oncogenic potential of HCC and might constitute a potential therapeutic target [21]. Lv et al. reported that HOXD9 overexpression could significantly enhance HCC cell migration, invasion and metastasis [22]. The associations of HOXD9, NDST3, PZP, E2F8, ADRA2B and COL15A1 with hepatocellular carcinoma were supported by clinical evidence from the Human Protein Atlas database (https://www.proteinatlas.org/).

There were several advantages in the present study. First, the Eight-mRNA prognostic nomogram could provide individual mortality risk assessment without the use of complex formula, which was convenient for patients without medical knowledge. Second, the Eight-mRNA prognostic nomogram could provide individual mortality risk assessment of 1-year, 2-year and 3-year overall survival. The individual survival predictions for different endpoints were important for patients to undertake timely treatments according to their mortality risk probability. Third, the results of the Eight-mRNA prognostic nomogram provided individual risk probability, which was easy to understand for patients without medical knowledge. Fourth, the potential prognostic mRNA biomarkers were identified by the LASSO Cox regression method, which is a popular method for high-dimensional data. Fifth, for HCC patients unwilling to undergo surgery or unable to tolerate surgery, the Eight-mRNA prognostic nomogram was an alternative noninvasive detection method and was more suitable for preoperative prediction.

The present study has several limitations that must be taken into account for clinical application. First, although we validated the clinical utility of the Eight-mRNA prognostic nomogram with a validation cohort from the GEO database, the Eight-mRNA prognostic nomogram lacks a prospective cohort study. Further clinical studies are needed to validate the clinical utility of the Eight-mRNA prognostic nomogram for HCC patients. Second, we screened the TCGA dataset and found eight mRNA biomarkers as predictors for the overall survival of HCC patients. The associations and impact mechanisms of these mRNAs for the overall survival of HCC patients have not yet been elucidated. Therefore, prospective experimental studies with a large sample size are needed to provide convincing evidence for the clinical application of the Eight-mRNA prognostic nomogram. Third, as a survival cohort study, some patients in the survival group were lost to follow-up and lack of insufficient survival data, which might influence the reliability of the results. Therefore, prospective survival cohort studies with a long follow-up observation will be helpful to provide high-level evidence for the overall survival of HCC patients. Fourth, as a template for protein synthesis, mRNAs are easily degradable, which may weaken the reliability of the conclusions of this research. Therefore, it is necessary to further validate the clinical utility of the present prognostic model by proteome studies before clinical application.

## Conclusion

In conclusion, the current study developed two convenient and efficient predictive precision medicine tools for hepatocellular carcinoma. These two predictive precision medicine tools are helpful for predicting the individual mortality risk probability and improving the personalized comprehensive treatments for HCC patients. The Smart Cancer Predictive System can be used by clicking the following URL:

https://zhangzhiqiao2.shinyapps.io/Smart_cancer_predictive_system_HCC_2/. The Gene Survival Analysis Screen System is available at the following URL: https://zhangzhiqiao5.shinyapps.io/Gene_Survival_Analysis_A1001/.

## Supplementary information

**Additional file 1.** Study flowchart. TCGA, The Cancer Genome Atlas.

**Additional file 2.** Heat map of the differential expression of mRNAs between 377 cancer samples and 50 adjacent normal tissues.

**Additional file 3.** Volcano plot of the differential expression of mRNAs between 377 cancer samples and 50 adjacent normal tissues.

## Data Availability

All related documents and data in the present study are available in the additional documents. Smart Cancer Predictive System can be used by clicking the following URL:https://zhangzhiqiao2.shinyapps.io/Smart_cancer_predictive_system_HCC_2/. Gene Survival Analysis Screen System is available at the following URL: https://zhangzhiqiao5.shinyapps.io/Gene_Survival_Analysis_A1001/.

## Abbreviations

HCC: hepatocellular carcinoma
TCGA: The Cancer Genome Atlas
ROC: receiver operating characteristic
OS: overall survival
mRNA: messenger RNA
HR: hazard ratio
CI: confidence interval
AJCC: The American Joint Committee on Cancer
SD: standard deviation
