# Supplementary figures and images for: Two predictive precision medicine tools for hepatocellular carcinoma
Source: Cancer Cell Int. 2019 Nov 14;19:290. doi: 10.1186/s12935-019-1002-z (PMC6854692; doi:10.1186/s12935-019-1002-z)

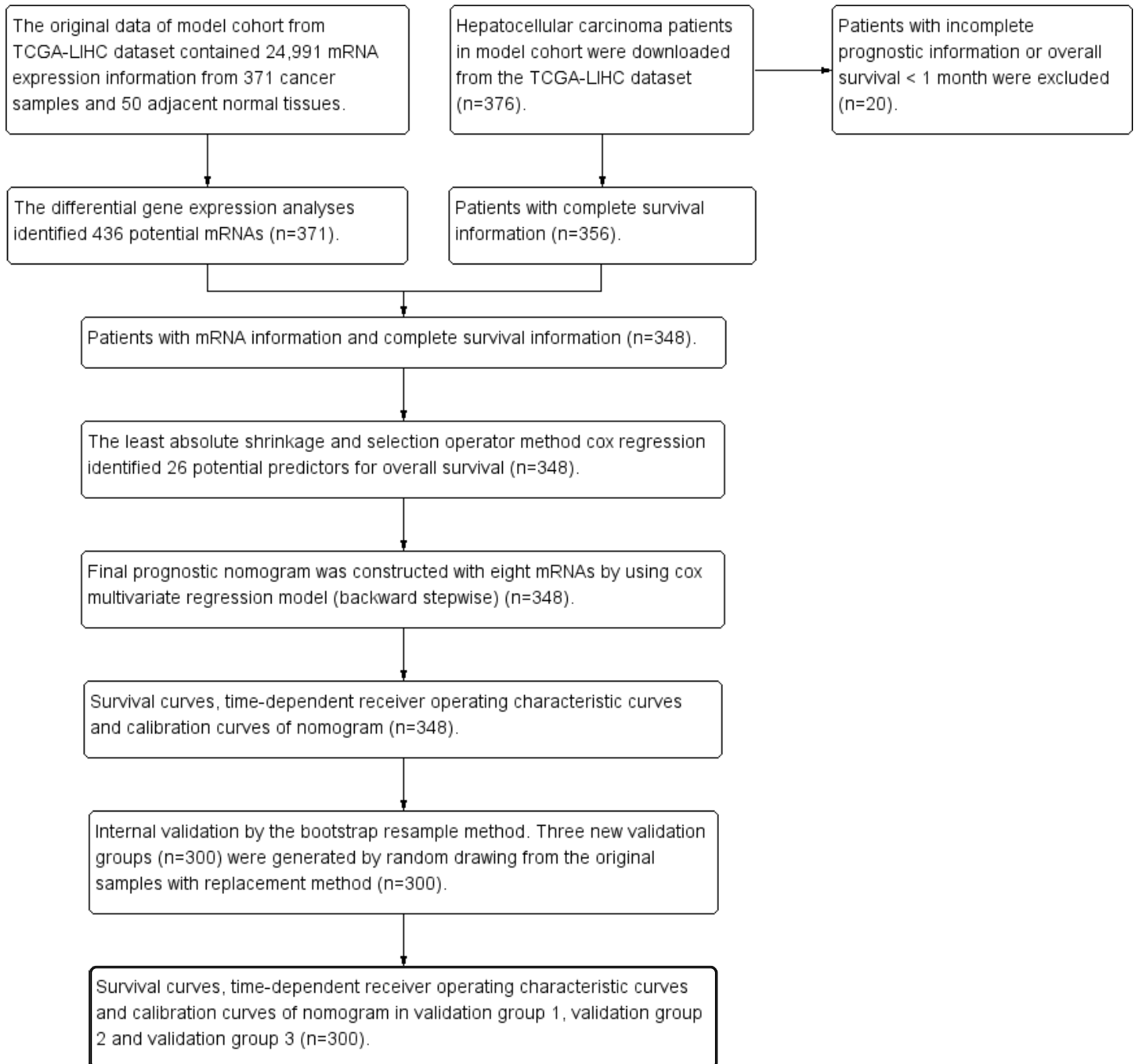

Supplement: Supplementary file 1 — Additional file 1. Study flowchart. TCGA, The Cancer Genome Atlas. [file 12935_2019_1002_MOESM1_ESM.pdf]

# Volcano

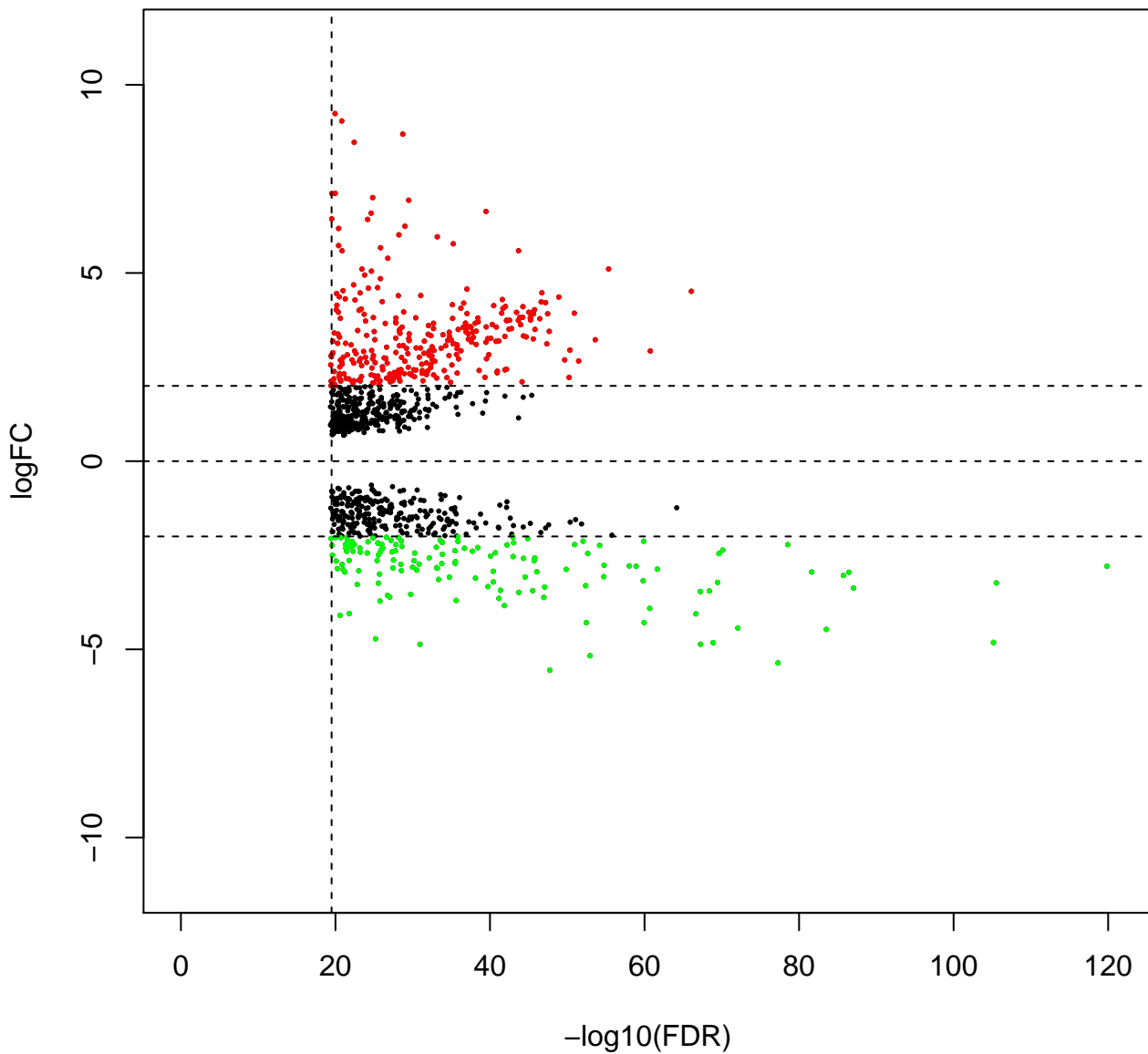

Supplement: Supplementary file 3 — Additional file 3. Volcano plot of the differential expression of mRNAs between 377 cancer samples and 50 adjacent normal tissues. [file 12935_2019_1002_MOESM3_ESM.pdf]
