# Supplementary material for: Two predictive precision medicine tools for hepatocellular carcinoma
Source: Cancer Cell Int. 2019 Nov 14;19:290. doi: 10.1186/s12935-019-1002-z (PMC6854692; doi:10.1186/s12935-019-1002-z)
Supplement: Supplementary file 2 — Additional file 2. Heat map of the differential expression of mRNAs between 377 cancer samples and 50 adjacent normal tissues. [file 12935_2019_1002_MOESM2_ESM.pdf]

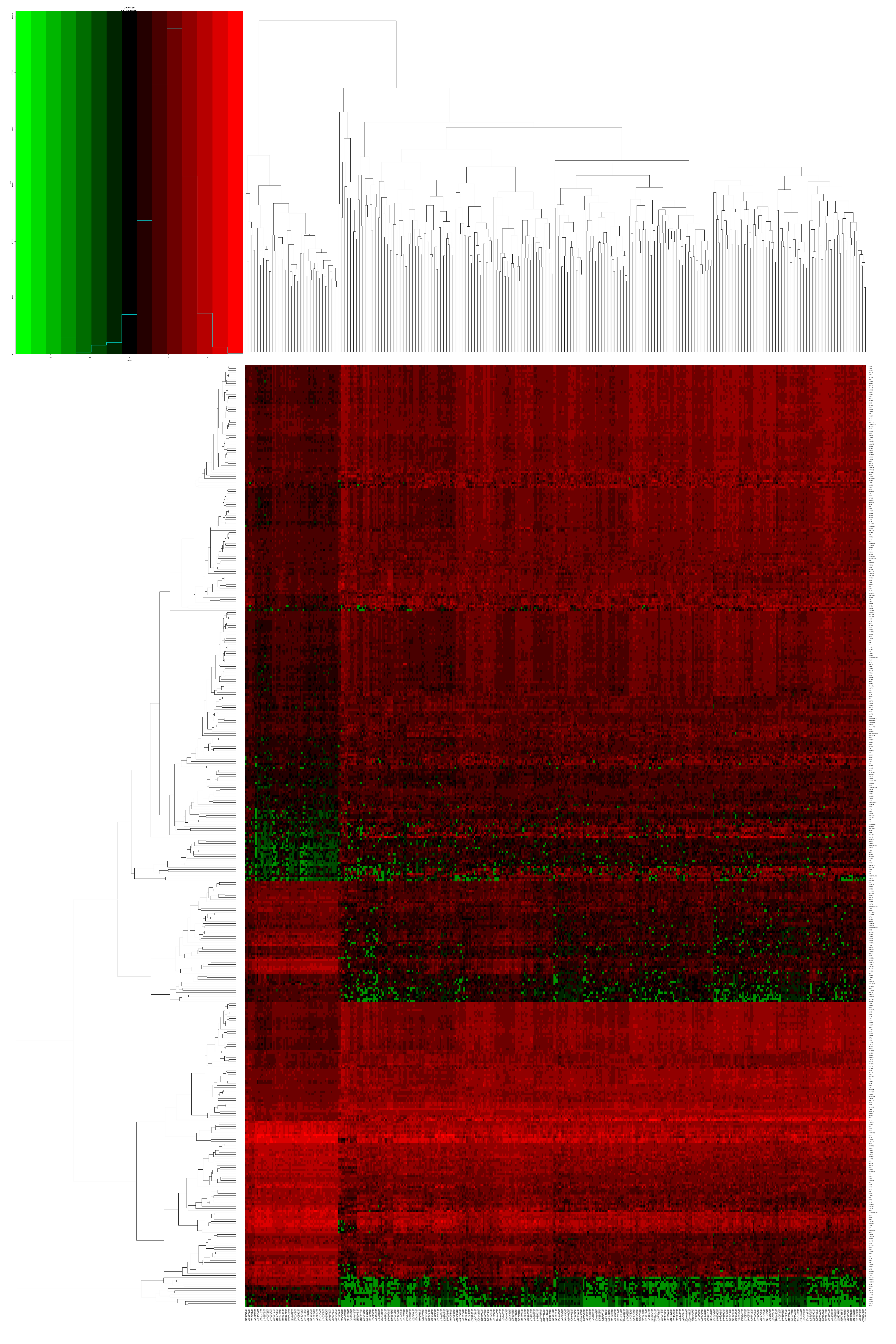
Color Key
and Histogram
Count
0
10000
20000
30000
40000
50000
60000
−4
−2
0
2
4
Value
